# Signatures of Conical Intersection Dynamics in the Time-Resolved Photoelectron Spectrum of Furan: Theoretical Modeling with an Ensemble Density Functional Theory Method

**DOI:** 10.3390/ijms22084276

**Published:** 2021-04-20

**Authors:** Michael Filatov, Seunghoon Lee, Hiroya Nakata, Cheol-Ho Choi

**Affiliations:** 1 Department of Chemistry, Kyungpook National University, Daegu 702-701, Korea; 2 Division of Chemistry and Chemical Engineering, California Institute of Technology, Pasadena, CA 91125, USA; slee89@caltech.edu; 3 R & D Center Kagoshima, Kyocera, 1-4 Kokubu Yamashita-cho, Kirishima-shi, Kagoshima 899-4312, Japan; hiroya.nakata.gt@kyocera.jp

**Keywords:** non-adiabatic dynamics, conical intersection, time-resolved photoelectron spectra, ionization potential, ensemble DFT

## Abstract

The non-adiabatic dynamics of furan excited in the ππ* state (S_2_ in the Franck–Condon geometry) was studied using non-adiabatic molecular dynamics simulations in connection with an ensemble density functional method. The time-resolved photoelectron spectra were theoretically simulated in a wide range of electron binding energies that covered the valence as well as the core electrons. The dynamics of the decay (rise) of the photoelectron signal were compared with the excited-state population dynamics. It was observed that the photoelectron signal decay parameters at certain electron binding energies displayed a good correlation with the events occurring during the excited-state dynamics. Thus, the time profile of the photoelectron intensity of the K-shell electrons of oxygen (decay constant of 34 ± 3 fs) showed a reasonable correlation with the time of passage through conical intersections with the ground state (47 ± 2 fs). The ground-state recovery constant of the photoelectron signal (121 ± 30 fs) was in good agreement with the theoretically obtained excited-state lifetime (93 ± 9 fs), as well as with the experimentally estimated recovery time constant (ca. 110 fs). Hence, it is proposed to complement the traditional TRPES observations with the trXPS (or trNEXAFS) measurements to obtain more reliable estimates of the most mechanistically important events during the excited-state dynamics.

## 1. Introduction

Time-resolved (TR) photoelectron (PE) spectroscopy (TRPES) is a pump–probe technique that is widely used to study the dynamics of photochemical reactions [1,2,3]. During the TRPE spectrum acquisition, a molecule is brought into an excited electronic state by the pump pulse and is subsequently probed by a series of short probe pulses, which cause ionization of the molecule and emanation of the photoelectrons [1,2,3]. In different experimental setups, the energy of the probe pulse varies from as little as a few (4–5) eV [4,5,6] to a much stronger ionizing radiation (∼14–30 eV) [7,8,9]; the latter covers the whole observation window for the valence electrons. Reaching the subvalence shells requires higher photon energies [10,11], and deeper core shells can be accessed with the time-resolved X-ray photoelectron spectra (trXPS) [12,13] or the transient near-edge X-ray absorption fine structure (trNEXAFS) [14,15] methods.

Furan is one of the popular objects for TRPES experiments [4,5,6,9], as it represents a prototypical example of a five-membered heterocyclic molecule. The UV absorption spectrum of furan is dominated by a broad band at ca. 6 eV (200 nm), which corresponds to a valence π→$\pi^{*}$ transition [4,16]. The lowest-energy Rydberg excited state occurs just a notch below the ${}^{1}$B${}_{2}$($\pi \pi^{*}$) valence excited state at ca. 5.91 eV [16]. According to TRPE spectroscopy [9] and TRPE imaging [4,5] measurements, when excited in the ${}^{1}$B${}_{2}$ (S${}_{2}$) state, furan directly undergoes an internal conversion to the ground state, and the Rydberg state is not populated. The non-adiabatic relaxation to the ground state may occur through two main reaction channels: the ring-puckering channel, which involves substantial deformation of the furan ring and may result in electrocyclization, and the ring-opening channel, which results in the formation of a number of products [4,5,6,9,17,18]. Theoretical simulations based on the complete active-space self-consistent field (CASSCF) method predicted that both reaction channels become active during the photoreaction [19,20]. Simulations based on time-dependent density functional theory (TDDFT) computations yielded a strong preference for the ring-puckering channel [4,5,9]. Although TDDFT is not appropriate for non-adiabatic molecular dynamics simulations, as it fails to correctly describe the topology of conical intersections [21,22,23], the latter simulations seemed to agree with the most recent TRPES measurements by Adachi et al. [9], which assigned only a minor fraction of the reaction products to the ring-opening channel.

Based on their TRPES measurements, Adachi et al. [9] concluded that the non-adiabatic transitions through conical intersections with the ground state manifest themselves in the form of a kink in the crest of the PE intensity at ca. 90 fs after the photoreaction starts. This identification was also supported by the TDDFT theoretical simulations. However, the latter methodology was not capable of properly describing conical intersections and its predictions should be accepted with skepticism, especially regarding the events of passage through conical intersections.

Hence, in this work, we attempt to revisit the theoretical analysis of the photodynamics of furan excited in the S${}_{2}$ ($\pi \pi^{*}$) state. The main purpose of this study is to investigate possible manifestations of the transitions through conical intersections in the TRPE spectra. To this end, we employ a computational methodology, the spin-restricted ensemble-referenced Kohn–Sham (REKS) method [24,25] in its state-interaction state-averaged (SI-SA-REKS, or SSR) variant [26,27,28,29], which is capable of describing the ground- and excited-state potential energy surfaces (PESs) of molecules, as well as the true crossings between these states, i.e., the conical intersections (CIs), correctly and accurately [22,23,27,30,31]. The SSR method will be used to carry out the non-adiabatic molecular dynamics (NAMD) simulations of furan excited in the $\pi \pi^{*}$ state and to theoretically model the resulting TRPE spectra in a wide range of electron binding energies (eBE). Modeling of the TRPE spectra will be carried out using the EKT-SSR method [32,33], which is capable of yielding the ionization energies and the respective Dyson orbitals of the ground and excited molecular electronic states.

In addition to identifying possible manifestations of the dynamics through conical intersections in TRPE spectra, the present work will address yet another important question: What time evolution characteristics of TRPE spectra can provide the closest estimates of the decay constant of the excited-state population (i.e., the excited-state lifetime)? Indeed, the valence ionization potentials and the respective Dyson orbitals in the excited state are very sensitive to the geometric changes that the molecule undergoes during the dynamics [34,35]. Hence, the decay of the PE intensity at the given eBE may occur at a faster rate than the population decay [34,35]. In recent theoretical simulations of the excited-state dynamics and the TRPE spectra of cyclohexadiene [35,36], it was observed that the rate of recovery of the ground-state PE intensity may provide a better estimate of the excited-state lifetime. Here, we shall test this observation for the excited-state reaction of furan.

In the following, we shall briefly outline the methodologies used in this work for the NAMD simulations. Then, the adiabatic PESs of the ground and excited states of furan will be investigated to elucidate possible reaction channels. The static computations will be followed by NAMD simulations and modeling of the TRPE spectra.

## 2. Computational Methods

### 2.1. SSR Method

In this work, the SSR method [24,25,26,27,28,29] is used to obtain the energies of the ground and excited electronic states, forces on the nuclei (the analytic gradient), and the non-adiabatic coupling (NAC) vector, as well as the molecular ionization energies and the respective Dyson orbitals. The SSR method has been described in the literature, and only the salient features of the method are given below.

The SSR method is based on ensemble density functional theory (eDFT), where the ground-state eDFT [37,38,39,40,41,42] is used to describe the non-dynamic electron correlation originating in multi-reference ground states of molecules and eDFT for ensembles of the ground and excited states [43,44,45,46] to obtain excitation energies from a variational time-independent computation. The ensemble representation of the density and the energy of a multi-reference electronic state results in the occurrence of the fractional occupation numbers (FONs) of several frontier KS orbitals [47,48,49]. Currently, the REKS/SSR methods have been implemented for systems with two [24,25,26,28,29] or four [50,51,52] fractionally occupied KS orbitals containing, in total, two or four active electrons, respectively; i.e., the (2,2) and (4,4) active spaces. These active spaces are sufficient for describing the dissociation of single and double chemical bonds; however, further extension of the methodology to larger active spaces is possible along the guidelines outlined in [50].

**Scheme 1:**
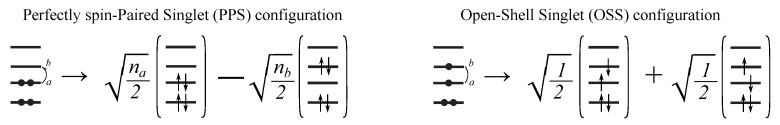
Schematic representation of the electronic configurations included in the SSR(2,2) method.

In the SSR method, the Gross–Oliveira–Kohn (GOK) variational principle [43,44,45,46] for ensembles of the ground and excited electronic states is implemented in connection with the charge-neutral excitations. In the case of the (2,2) active space, the ground state is approximated by a perfectly spin-paired singlet (PPS) configuration and an excited state by an open-shell singlet (OSS) configuration [50]; see Scheme 1. The fractional occupation numbers (FONs) of the active orbitals in the PPS configuration, labelled *a* and *b* in Scheme 1, are variationally optimized together with the KS orbitals for an ensemble of the PPS and OSS configurations (Equation (1)):

(1) $$\begin{array}{cc} E^{SA-REKS}= & w_{PPS}E_{0}^{PPS}+w_{OSS}E_{1}^{OSS}\\ \; & w_{PPS}+w_{OSS}=1 \end{array}$$

where the subscripts 0 and 1 are added to underline that the PPS configuration is regarded as the ground state and the OSS configuration as an excited state. The open-shell self-consistent field (SCF) methodology [53] is used for the orbital optimization.

The SCF optimization of the orbitals and their FONs is followed by solving a 2 × 2 secular problem,

(2) $$\left(\begin{array}{cc} E_{0}^{PPS} & \Delta_{01}^{SA}\\ \Delta_{01}^{SA} & E_{1}^{OSS} \end{array}\right)\left(\begin{array}{cc} a_{00} & a_{01}\\ a_{10} & a_{11} \end{array}\right)=\left(\begin{array}{cc} E_{0}^{SSR} & 0\\ 0 & E_{1}^{SSR} \end{array}\right)\left(\begin{array}{cc} a_{00} & a_{01}\\ a_{10} & a_{11} \end{array}\right)$$

to include possible couplings between the PPS and the OSS electronic configurations. In Equation (2), the coupling element $\Delta_{01}^{SA}$

(3) $$\Delta_{01}^{SA}=\left(\sqrt{n_{a}}-\sqrt{n_{b}}\right)\varepsilon_{ab}^{SA}$$

is calculated using the FONs $n_{a}$ and $n_{b}$ of the active orbitals and the Lagrangian matrix element $\varepsilon_{ab}^{SA}$ between the active orbitals *a* and *b*, which are obtained during the SCF optimization of the SA-REKS functional (1) [27,29,50]. The solutions $E_{0}^{SSR}$ and $E_{1}^{SSR}$ of Equation (2) furnish the ground (S${}_{0}$) and excited (S${}_{1}$) state energies in the SSR(2,2) method.

In the SSR method, the GOK variational principle is used in connection with an ensemble of charge-neutral excitations. Hence, differentiation with respect to the ensemble weights, $w_{PPS}$ and $w_{OSS}$, recovers the excitation energy. This contrasts with some alternative formulations of eDFT-based methods [54,55], which operate with charged excitations, and differentiation with respect to the ensemble weights recovers the electron-addition/electron-removal energies. At variance with the single-reference SCF methods, such as the Hartree–Fock or the Kohn–Sham methods, the orbitals and the respective Lagrangian matrix in an open-shell SCF formalism [53], such as the SSR method, do not satisfy Koopmans’ theorem [56], and cannot be used for obtaining the ionization energies either.

Hence, the ionization energies are obtained in the SSR method through the use of the extended Koopmans’ theorem [57,58] (EKT), according to which they are represented by the eigenvalues and the eigenfunctions of the Lagrangian matrix expressed on the basis of the natural orbitals [57,58]. For the quantum chemical methods, for which the analytic energy gradient is available, the proper one-particle density matrix and the effective Lagrangian occur in the expression for the energy derivative with respect to the nuclear coordinates. Hence, the EKT ionization energies can be obtained at the level of correlated wavefunction theory methods [59,60]. In the EKT-SSR method, the (negatives of the) ionization energies $\varepsilon^{X}$ and the respective Dyson orbitals $C^{X}$ for the state *X* (= S${}_{0}$, S${}_{1}$) are obtained by solving a generalized eigenvalue problem

(4) $${\tilde{W}}^{X}\;C^{X}=D^{X}\;C^{X}\;\varepsilon^{X}\;,$$

where $D^{X}$ is the relaxed density matrix for the state *X* and ${\tilde{W}}^{X}$ is the Lagrangian matrix that occurs in the expression for the analytic energy gradient of the individual state *X* [32,52]. If a single-reference SCF method is used for obtaining the density and the Lagrangian matrices in Equation (4), it would become fully equivalent to the usual Koopmans’ theorem. In the case of SSR, the $D^{X}$ and ${\tilde{W}}^{X}$ matrices include the orbital response contributions, which are obtained by solving the coupled-perturbed REKS (CP-REKS) equations [32,52].

The (squares of the) norms of the Dyson orbitals $C^{X}$

(5) $$\left|\right|\gamma^{X}{\left|\right|}^{2}=tr\left({\left(C^{X}\right)}^{†}\;D^{X}\;{\left(D^{X}\right)}^{†}\;C^{X}\right)\;.$$

yield the pole strengths of the respective ionizations [59]. The probability of the ionization is proportional to the respective Dyson norm when a transition of the ionized electron into an unstructured continuum of states occurs [61]. In the present work, the norms of Dyson’s orbitals are used as proxies of the ionization intensities when modeling the PE and TRPE spectra of the molecules studied here. For the molecules in their respective electronic states X (= S${}_{0}$, S${}_{1}$), the intensity of the PES signal $I_{PES}^{X}\left(\varepsilon \right)$ at the eBE ε is calculated using the ionization energies $-\varepsilon_{j}^{X}$ and the norms $|\gamma_{j}^{X}{|}^{2}$ of the respective Dyson orbitals obtained with Equations (4) and (5),

(6) $$\begin{array}{cc} I_{PES}^{X}\left(\varepsilon \right) & \propto \sum_{K}\sum_{j}{\left|\gamma_{j}^{X}\left(R_{K}\right)\right|}^{2}\;f_{L}\left(\varepsilon ;-\varepsilon_{j}^{X}\left(R_{K}\right)\right)\\ f_{L}\left(\varepsilon ;\varepsilon_{0}\right) & =\frac{\lambda_{\varepsilon }}{\pi }\cdot \frac{1}{1+{\left(\frac{\varepsilon -\varepsilon_{0}}{\lambda_{\varepsilon }}\right)}^{2}}, \end{array}$$

at the geometries $R_{K}$ that are obtained, e.g., by sampling the Wigner function [62,63]. The individual lines are convoluted using the Lorentzian lineshape function $f_{L}$, where $\lambda_{\varepsilon }$ is the half width at half maximum (HWHM). The lineshape function replaces the δ function $\delta (\varepsilon -eBE_{j})$, where ε is the energy of the ionizing probe photons and $eBE_{j}$ is the electron binding energy of the *j*-th ionization channel.

The intensity of the TRPES signal at a time instance τ is obtained as a convolution (with the 2D Lorentzian lineshape function) of the ionization energies and the respective Dyson norms calculated at instantaneous geometries $R_{K}\left(t_{i}\right)$ from the trajectory *K* in the electronic state *X* (= S${}_{0}$, S${}_{1}$) at time instance $t_{i}$ [64]:

(7) $$\begin{array}{cc} \; & I_{TRPES}\left(\varepsilon ;\tau \right)\propto \sum_{K}^{N_{traj}}\sum_{X(=S_{0},S_{1})}\sum_{j}\sum_{i}^{N_{steps}}N_{X,K}\left(t_{i}\right){\left|\gamma_{j}^{X}\left(R_{K}\left(t_{i}\right)\right)\right|}^{2}\times \\ \; & \;\;\;f_{L,2D}\left(\varepsilon ,\tau ;-\varepsilon_{j}^{X}\left(R_{K}\left(t_{i}\right)\right),t_{i}\right)\;;\\ \; & f_{L,2D}\left(\varepsilon ,t;\varepsilon_{0},t_{0}\right)=\frac{\lambda_{\varepsilon }\;;\lambda_{t}}{\pi^{2}}\cdot \frac{1}{1+{\left(\frac{\varepsilon -\varepsilon_{0}}{\lambda_{\varepsilon }}\right)}^{2}}\cdot \frac{1}{1+{\left(\frac{t-t_{0}}{\lambda_{t}}\right)}^{2}}\;, \end{array}$$

where $N_{X,L}\left(t_{i}\right)$ is the population of the state *X* in trajectory *K* at time $t_{i}$. The 2D Lorentzian function $f_{L,2D}$ replaces the respective δ function and introduces broadening along the energy axis, as well as the time axis. The latter broadening ensures that $I_{TRPES}\left(\varepsilon ;\tau \right)$ remains a continuous function of time τ, although the ionization energies and intensities are obtained at discrete time instances $t_{i}$.

### 2.2. Computational Details

The computations reported here were performed using a locally modified version of the GAMESS-US program [65,66], which implemented the SSR(2,2) method and its analytical derivative formalism [67], including the SSR-EKT methodology for obtaining the ionization potentials [32] and the electron affinities [33] of the ground and excited states of molecules. The calculations employed the 6-311G** basis set [68] and the BH&HLYP density functional [69,70,71]. The geometry optimizations were performed using the standalone version of the DL-FIND program [72]. The minimum energy paths were optimized using the nudged elastic band (NEB) method [73], which was implemented in DL-FIND. The geometries of the conical intersections were optimized by the CIOpt program [74] with the penalty function formalism and by using the analytic energy gradients of the intersecting states.

The NAMD simulations were performed by the UNI-xMD program, a standalone code that implemented the DISH-XF method [75]. The DISH-XF method combined the electronic equations derived from the exact factorization of the electronic–nuclear wavefunction [76,77,78,79,80] with the conventional trajectory surface hopping (TSH) formalism [81]. The exact factorization enabled seamless incorporation of the effect of nuclear quantum momentum, which depends on the shape of the nuclear distribution, into the classical equations of motion for the nuclei.

Here, the nuclear equations of motion were integrated using the velocity–Verlet algorithm with the time step of 10 a.u. (0.24 fs). The initial conditions at the start of the NAMD trajectories were set up by sampling the Wigner function of a canonical ensemble [62,63] at T = 300 K. The trajectories were propagated for 1000 steps (240 fs) using the NVE ensemble, i.e., the microcanonical ensemble, where the total number of particles *N*, the system’s volume *V*, and the total (electronic + nuclear kinetic) energy *E* were conserved.

The geometries obtained by sampling the Wigner function were used to construct the PE spectra according to Equation (6) with HWHM $\lambda_{\varepsilon }$ = 0.1 eV. When modeling the TRPE spectra with Equation (7), the same value of the energy broadening was used. The values of the ionization energies and intensities were recorded with a time interval of 2.4 fs, and the time-broadening parameter $\lambda_{t}$ (see Equation (7)) was set to 1.2 fs.

## 3. Results and Discussion

Before presenting the results of the NAMD simulations of the excited furan and its simulated PE and TRPE spectra, the mechanism of the reaction will be briefly discussed in the following. As mentioned in the introduction, upon the excitation in the $\pi \pi^{*}$ (S${}_{2}$ at the Franck–Condon (FC) geometry) state, no internal conversion in the Rydberg π3s (S${}_{1}$ at FC) state was detected experimentally [4,5,9]. Hence, when computing the stationary points on the ground- and excited-state potential energy surfaces (PESs) and when modeling the dynamics, no attempt was made to include the Rydberg states. According to the previous theoretical computations [6,18], the S${}_{2}$/S${}_{1}$ crossing occurs in the vicinity of the FC geometry, and the $\pi \pi^{*}$ state becomes the S${}_{1}$ adiabatic state.

### 3.1. Stationary Points and Adiabatic Minimum Energy Paths (MEPs) on the Ground- and Excited-State PESs of Furan

**Scheme 2:**
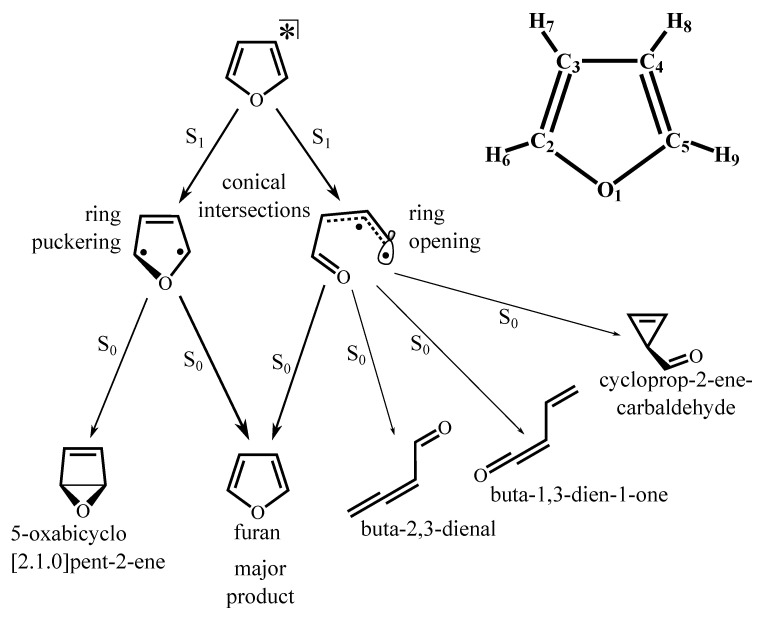
Schematic representation of the excited-state deactivation mechanism of furan. The reaction products shown in the scheme are cited from [4,9]. The inset in the upper-right corner shows the atomic numbering used in this work. The electronically excited furan is labelled by an asterisk.

**Figure 1:**
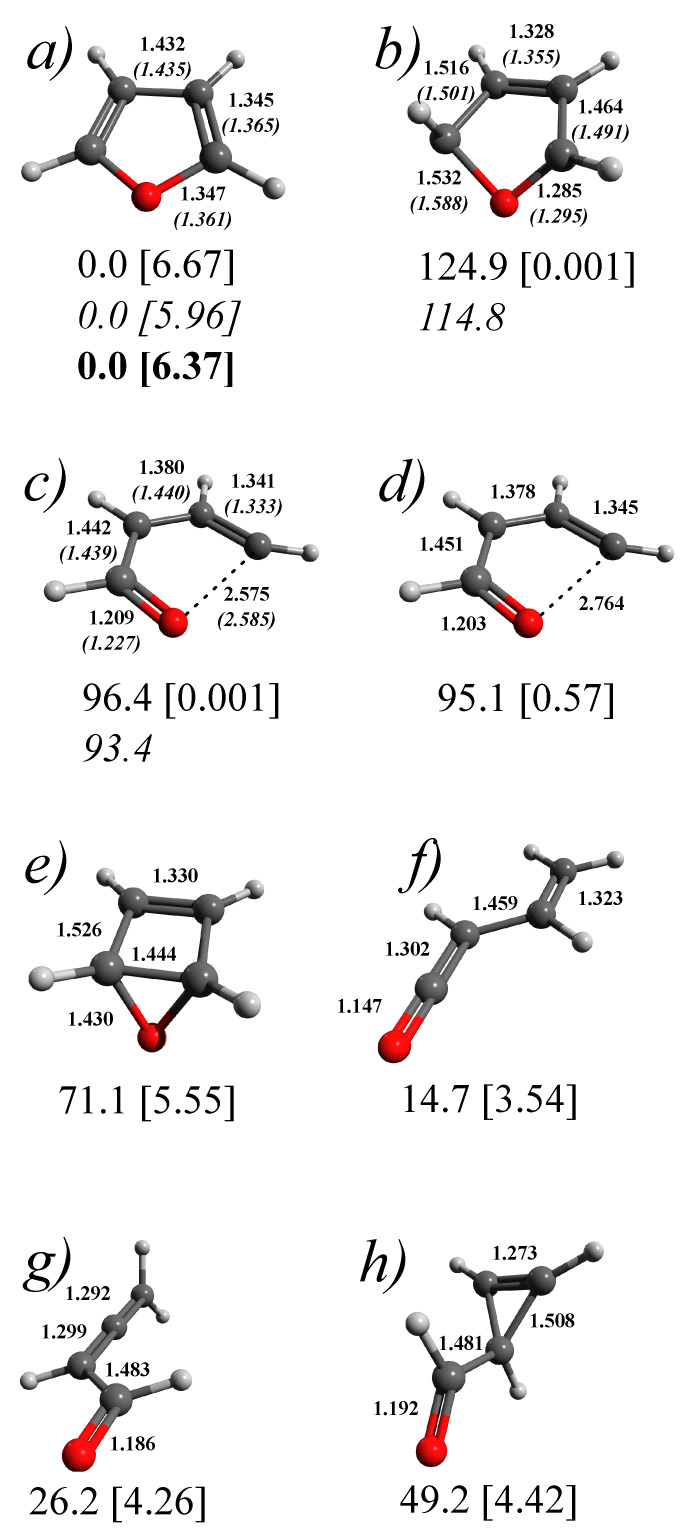
Geometries of the S${}_{0}$ stationary points and their relative energies optimized using the SSR-BH&HLYP/6-311G** method. The key bondlengths (Å) given in normal font were obtained in this work. The italicized bondlengths were obtained with the (MS)CASPT2/6-311G* method in [18]. The relative energies (kcal/mol, below the geometries) given in normal font were obtained in this work. The italicized energies are cited from [18]. In square brackets, the vertical excitation energies (eV) at the given geometry are shown: normal font—this work, italicized—Ref. [18], boldface—the TBE value from [82]. The following compounds and geometries are shown in Figure 1: (**a**) furan (S${}_{0}$ minimum), (**b**) CI${}_{\mathrm{puck}}$, (**c**) CI${}_{\mathrm{ropn}}$, (**d**) but-2-enal-4-ylidene (S${}_{0}$), (**e**) 5-oxabicyclo[2.1.0]pent-2-ene (S${}_{0}$), (**f**) buta-2,3-dienal (S${}_{0}$), (**g**) buta-1,3,-dien-1-one (S${}_{0}$), and (**h**) cycloprop-2-ene-carbaldehyde (S${}_{0}$).

According to the prior theoretical results [4,9,18], deactivation of the furan excited in the $\pi \pi^{*}$ state occurs via two possible paths, as shown in Scheme 2. The first path involves puckering of the furan ring and proceeds through the ring-puckering conical intersection (CI) CI${}_{\mathrm{puck}}$ between the S${}_{1}$ and S${}_{0}$ states; see Figure 1 for the geometry. This path leads to either the recovery of the initial conformation or the formation of a bicyclic compound, 5-oxabicyclo[2.1.0]pent-2-ene, by closing a single bond between the C${}_{2}$ and C${}_{5}$ atoms. The second reaction path proceeds through the ring-opening CI, CI${}_{\mathrm{ropn}}$, and can result in the formation of a variety of products. According to the previous theoretical simulations [4,9], the formation of these products occurs on the ground-state PES after relaxing through one of the CIs. As the molecule remains sufficiently “hot” upon the S${}_{1}$→ S${}_{0}$ relaxation, the excess vibrational energy assists in overcoming the respective energy barriers.

The equilibrium geometries of the species shown in Scheme 2 were obtained using the SSR-BH&HLYP/6-311G** method and are shown in Figure 1. For comparison, the lengths of the key bonds obtained by Stenrup and Larson [18] using the (MS)CASPT2/6-311G* method are shown parenthetically in Figure 1. The relative energies (in kcal/mol) of the two mechanistically important CIs, CI${}_{\mathrm{puck}}$ and CI${}_{\mathrm{ropn}}$, obtained in this work are in reasonable agreement with the (MS)CASPT2/6-311G* energies from [18].

The vertical excitation energies (VEE, in eV) at the respective geometries are shown in square brackets in Figure 1. At the S${}_{0}$ equilibrium geometry of furan, the VEE obtained in this work is 6.67 eV, which is slightly overestimated as compared to the theoretical best estimate (TBE) value of 6.37 eV [82]. Interestingly, in the vicinity of CI${}_{\mathrm{ropn}}$, there is a shallow minimum on the S${}_{0}$ PES shown in Figure 1d. This local minimum (but-2-enal-4-ylidene) has the electronic structure of a carbene biradical, the ground state of which can be represented by a PSS configuration, $C_{n}|n\bar{n}\rangle -C_{\pi }|\pi \bar{\pi }\rangle$, with the (lone-pair) *n* orbital located on the terminal C${}_{5}$ atom and the π orbital delocalized over the (allyl) C${}_{3}$–C${}_{4}$–C${}_{5}$ fragment. As a very unstable and reactive species, but-2-enal-4-ylidene can rearrange in one of the reaction products shown in Scheme 2 by either closing the ring at the O${}_{1}$ or C${}_{3}$ positions or through a hydrogen shift. Although the ground-state reaction paths leading to the formation of the final products may be quite interesting, their investigation is beyond the scope of the present work. In the following, we shall focus on the excited-state reaction paths and the dynamics of the excited-state relaxation.

**Figure 2:**
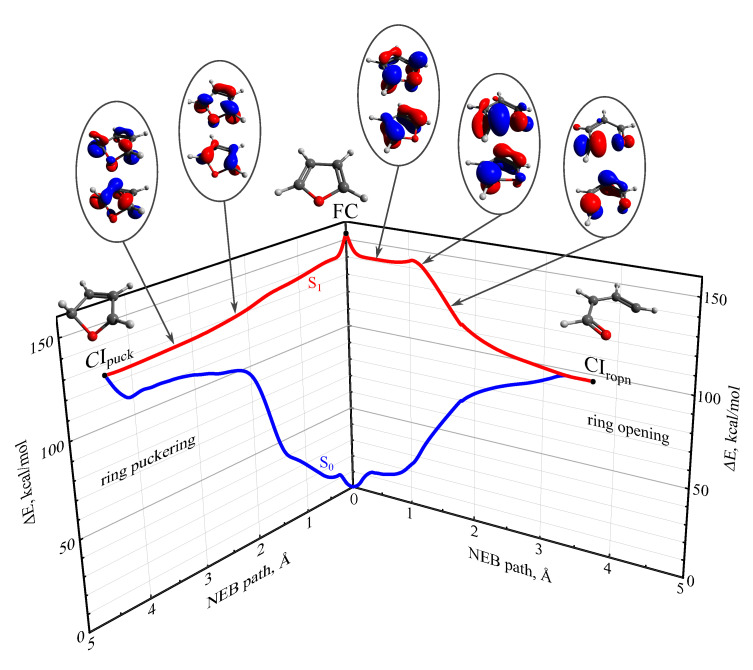
Minimum energy paths (MEPs) connecting the FC point in the $\pi \pi^{*}$ excited state with the ring-puckering and ring-opening CIs, CI${}_{\mathrm{puck}}$ and CI${}_{\mathrm{ropn}}$, respectively. The MEPs were optimized in the excited state (the red line) using the NEB method. The S${}_{0}$ state energies calculated at the NEB geometries are shown by the blue line. The abscissa axis gives the NEB path length (Å). The insets show the frontier Dyson orbitals obtained at the indicated geometries in the $\pi \pi^{*}$ excited state.

Using the S${}_{0}$ equilibrium geometry of furan (the Franck–Condon (FC) geometry) and the geometries of the two conical intersections, CI${}_{\mathrm{puck}}$ and CI${}_{\mathrm{ropn}}$, two minimum energy paths (MEPs) were optimized on the $\pi \pi^{*}$ excited-state PES. The two MEPs are shown in Figure 2, where the red lines show the MEPs optimized on the $\pi \pi^{*}$ excited-state PES and the blue lines show the (vertically de-excited) S${}_{0}$ energies at these geometries. The ring-puckering MEP does not feature any barriers, and the CI${}_{\mathrm{puck}}$ geometry is easily reached by sliding ca. 30 kcal/mol down the surface from 153.8 (FC point) to 124.9 kcal/mol (CI). CI${}_{\mathrm{puck}}$ features a non-symmetric geometry reached in an uneven disrotatory fashion by torsion about the C${}_{2}$–C${}_{3}$ and C${}_{4}$–C${}_{5}$ bonds accompanied by an out-of-plane displacement of the O${}_{1}$ atom. On the way from the FC point to CI${}_{\mathrm{puck}}$, the two frontier Dyson orbitals of furan, which, in the $\pi \pi^{*}$ state, have fractional norms, gradually transform from the $a_{2}$-symmetric (in the C${}_{2v}$ group) and the $b_{1}$-symmetric orbitals into the in-phase and out-of-phase combinations of the *p*-type orbitals of the C${}_{2}$ and C${}_{5}$ atoms. However, the electronic structure of both the ground and the excited states in the vicinity of CI${}_{\mathrm{puck}}$ does not become pure biradical, and is best described as a mixture of biradical and zwitterionic configurations, with the excited state being slightly more ionic.

The ring-opening CI${}_{\mathrm{ropn}}$ lies 28.5 kcal/mol lower than the ring-puckering CI${}_{\mathrm{puck}}$. However, the accessibility of this CI is hindered by a low barrier of ca. 2.7 kcal/mol on the excited-state PES; see Figure 2. In the vicinity of the FC geometry, the excited state along the ring-opening path retains its $\pi \pi^{*}$ characteristic. However, as the C–O bond is increasingly stretched, the $\pi^{*}$ orbital ($b_{1}$ under the C${}_{2v}$ symmetry) becomes mixed with the C–O $\sigma^{*}$ orbital. The gradual transformation of the respective Dyson orbitals from $\pi^{*}$ to $\sigma^{*}$ is shown in Figure 2. Hence, the low barrier on the excited-state PES results from mixing between the $\pi \pi^{*}$ and the $\pi \sigma^{*}$ electronic configurations. The occurrence of a barrier on the S${}_{1}$ PES is consistent with the results of the previous theoretical calculations [17,18]. For instance, Stenrup and Larson [18] and Gromov et al. [17] used the EOM-CCSD method and found a barrier on the ring-opening path of excited furan. The height of the barrier is, however, difficult to evaluate, as no proper geometry optimizations were carried out in these works. On the other hand, the CASSCF calculations performed by Hua et al. [19] and Oesterling et al. [20] found a barrierless ring-opening path. However, the CASSCF method lacks the contribution of the dynamic electron correlation, and its predictions regarding the activation barriers and chemical bond strengths should not be trusted. Hence, it is plausible to assume that the ring-opening path features a shallow barrier. The existence of a barrier is also supported by the experimental measurements by Adachi et al. [9].

**Figure 3:**
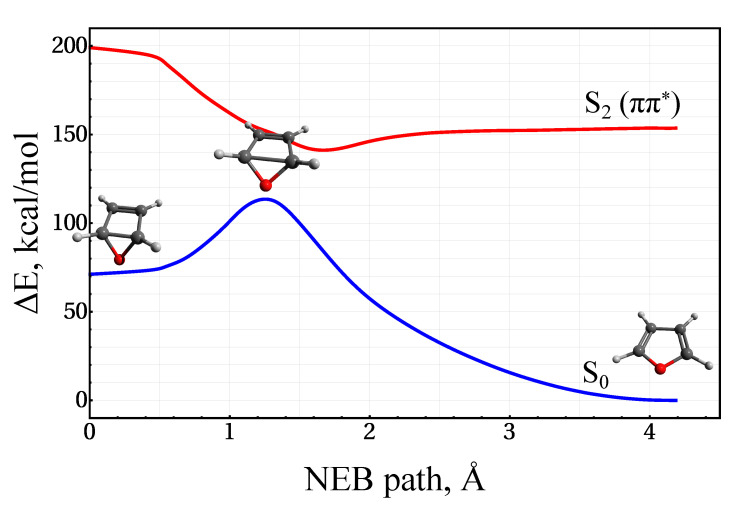
Minimum energy paths (MEPs) connecting the 5-oxabicyclo[2.1.0]pent-2-ene S${}_{0}$ equilibrium geometry with the S${}_{0}$ furan equilibrium geometry. The MEP was optimized in the S${}_{0}$ state using the NEB method. The blue line shows the S${}_{0}$ MEP. The red line shows the vertical projection of the S${}_{0}$ path onto the S${}_{2}$ ($\pi \pi^{*}$) PES.

The inspection of the MEPs in Figure 2 suggests that the ring-opening path is most likely hindered and the excited-state reaction should proceed predominantly along the ring-puckering path. Hence, one may expect that the main products of the furan photoreaction should be furan and 5-oxabicyclo[2.1.0]pent-2-ene. Although 5-oxabicyclo[2.1.0]pent-2-ene is the least stable of all the possible products shown in Figure 1, its ground-state minimum is separated from the furan S${}_{0}$ equilibrium geometry by a barrier of 42.0 kcal/mol (113.1 kcal/mol with respect to furan); see Figure 3. The sufficiently high barrier may prevent rapid decomposition of the thermodynamically unstable 5-oxabicyclo[2.1.0]pent-2-ene compound.

As regards possible products of the ring-opening path, it may be expected that small amounts of buta-2,3-dienal (1,3-butadienal) and buta-1,3-dien-1-one (1,2-butadienal, synonymous with 2,3-butadienal) may survive in the final mix of products. Formation of the final products, however, occurs in the ground electronic state, and its investigation is beyond the scope of the present work, which focuses on the modeling of the ultrafast non-adiabatic dynamics of the excited furan.

### 3.2. Static PE Spectra of Furan and the Photoproducts

As in the following, we shall focus on modeling the excited-state dynamics and the pertinent TRPE spectra, it seems appropriate to inspect the static PE spectra of the species shown in Figure 1 and to evaluate what transformations of these spectra may occur as a result of the photoreaction. Typically, the experimentally measured TRPE spectra are presented in the form of differential spectra, where the signal intensity at t<0, i.e., the ground-state PES, is subtracted from the measured intensity at the current *t*. Hence, we shall also discuss the S${}_{2}$− S${}_{0}$ difference in the PE spectra of furan, which can be compared with the bleach PE spectrum reported by Adachi et al. [9]. Modeling of the products’ PE spectra should assist in interpreting the changes that might be visible in the long-term limit of the TRPES observation.

The static PE spectra of the compounds in Figure 1 were simulated according to Equation (6). At each ground- and excited-state local minimum, one hundred geometries were generated by sampling the Wigner function of an ensemble of the harmonic oscillators at T = 300 K. (As a result of the photoreaction, hot photoproducts are typically formed; hence, their PE spectra should have been simulated at an elevated temperature. However, the dynamic simulations in this work employ the microcanonical (i.e., NVE) ensemble, and the temperature of the photoproducts is not defined. As their spectra were simulated at the same temperature (300 K) as for the reactants, one might expect a considerable broadening of the peaks in their PE spectra upon the photoreaction.) At each sampling geometry, the ionization energies, the Dyson orbitals, and their norms were calculated with the EKT-SSR-BH&HLYP/6-311G** method. As the common density functionals, such as the BH&HLYP, introduce a systematic shift in the ionization energies computed from the Koopmans’ or the extended Koopmans’ theorems [83,84,85], the computed ionization energies were shifted to the east by a uniform shift of 1.66 eV [32] to match the first ionization band maximum of furan’s PES reported by Adachi et al. [9]. The computed ionization lines were weighted by the respective Dyson norms and convoluted by the Lorentzian lineshape function with an HWHM of 0.1 eV.

**Figure 4:**
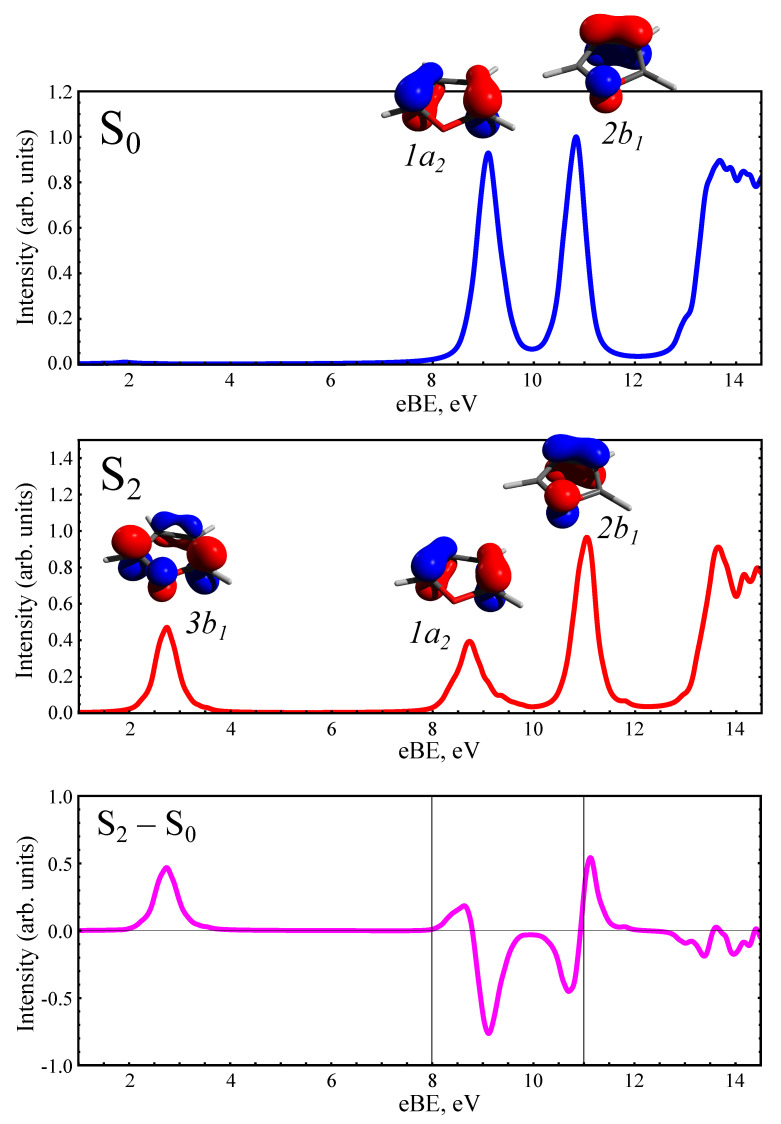
PE spectra of furan in the ground and the $\pi \pi^{*}$ (S${}_{2}$) excited states. All the spectra were normalized against the $2b_{1}$-band of the S${}_{0}$ furan. The lower panel shows the difference between the S${}_{2}$ and S${}_{0}$ spectra. The orbitals corresponding to the ionization bands are shown in the insets. The vertical lines in the lower panel show the observation window reported in [9].

As seen in Figure 4, the two lowest ionization bands of the S${}_{0}$ furan correspond to the $1a_{2}$ (at 9.1 eV) and $2b_{1}$ (at 10.8 eV) Dyson orbitals shown in the insets in the figure. The $2b_{1}$ orbital comprises the π-type oxygen lone pair and the C${}_{3}$–C${}_{4}$π-bonding orbital. The $1a_{2}$ orbital is an out-of-phase combination of the C${}_{2}$–C${}_{3}$ and C${}_{4}$–C${}_{5}$π-bonding orbitals. The computed ionization band maxima agree reasonably well with the vertical ionization potentials estimated from the He(I) PES of furan: 8.9 eV ($1a_{2}$) and 10.4 eV ($2b_{1}$) [86], respectively.

The π→$\pi^{*}$ excitation in the S${}_{2}$ state at the FC geometry populates the $3b_{1}$ symmetric orbital (see Figure 4), which comprises a $\pi^{*}$ orbital of the butadiene fragment C${}_{2}$–C${}_{3}$–C${}_{4}$–C${}_{5}$ and the π-type lone pair of oxygen. The corresponding maximum occurs at 2.7 eV. The π-type $1a_{2}$ orbital of furan is depopulated by the excitation, and its maximum shifts from 9.1 eV (S${}_{0}$) to 8.7 eV (S${}_{2}$). Both bands, $3b_{1}$ and $1a_{2}$, have a lower probability of ionization due to the fractional population of the respective natural orbitals [32,35]. The occurrence of a PE band at the electron binding energy (eBE) of 2.7 eV is consistent with the experimental observations. Thus, Fuji et al. [4] reported a weak-intensity band occurring at the eBE of ca. 3 eV at t=0 in their pump–probe (6.2 eV pump and 4.7 eV probe) photoelectron imaging experiments. The onset of a weak-intensity band at approximately the same eBE is also visible in the TRPE spectra reported by Adachi et al. [9], who used the 14 eV ionizing radiation. The latter authors also reported the PE bleach spectrum of furan (see Figure 4a of [9]), which is consistent with the difference PE spectrum shown in the lower panel of Figure 4; for the reader’s convenience, the observation window from [9] is shown by the vertical lines in Figure 4. Due to the approximation of the ionization probability by the norm of the Dyson orbitals, the lineshape of the ionization bands may come out somewhat different from in the experiments. However, there seems to be a general agreement with the experimentally observed PE spectra, which is important for the further modeling and interpretation of the TRPE spectra.

**Figure 5:**
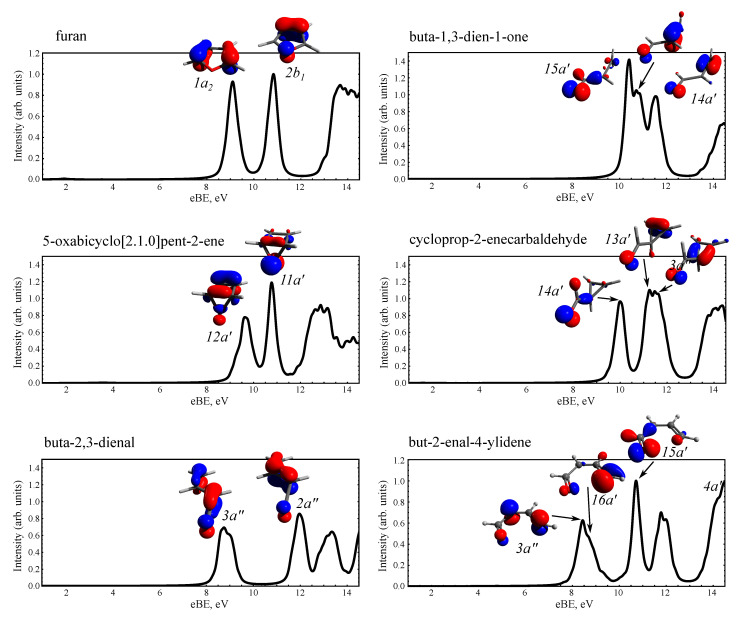
PE spectra of the ground-state species reported in Figure 1. The insets show the Dyson orbitals corresponding to the ionization bands.

The PE spectra of the ground-state species from Figure 1 are shown in Figure 5. The main purpose of displaying these spectra is to evaluate possible differences between the S${}_{0}$ PE spectrum of furan and the PE spectrum of a product mix. For instance, the PE spectrum of 5-oxabicyclo[2.1.0]pent-2-ene has the maximum of the first ionization band at 9.6 eV, i.e., shifted to the east by ca. 0.5 eV with respect to the first ionization band of S${}_{0}$ furan. Hence, the occurrence of 5-oxabicyclo[2.1.0]pent-2-ene in the final mix of photoproducts should result in a broadening of the first ionization band and a shift toward higher ionization energies. The same is true for buta-1,3-dien-1-one (the first band at 10.4 eV) and cycloprop-2-enecarbaldehyde (10 eV). However, the occurrence of sizable amounts of buta-2,3-dienal (the first band at 8.7 eV) should result in a shift to the lower ionization energies. As for but-2-enal-ylidene (the first band at 8.44 eV), its occurrence in the final product mix is not expected, as this is a very unstable species that should, most likely, undergo a quick rearrangement into other products. However, this molecule may be present at the early times of the photoreaction.

Adachi et al. [9] reported that after 1.5 ps from the start of the photoreaction, the PE spectrum of the product mix features a broad band between 9 and 9.5 eV and another broad band between 10 and 11 eV. The latter band features local maxima at ca. 10.2 and 10.7 eV; see Figure 4b of [9]. Hence, by comparing the reported long-term limit of the TRPE spectrum with the simulated PE spectra of the photoproducts, it can be conjectured that the dominant photoproducts may be the furan itself, the 5-oxabicyclo[2.1.0]pent-2-ene molecule, and, most likely, the buta-1,3-dien-1-one molecule; the latter occurs in the ring-opening channel. The exact ratio of the products is, however, difficult to evaluate on the basis of this comparison. Adachi et al. [9] argued that, as the intensity of the TRPES signal at eBE = 9.1 eV recovers only ca. 91% (not 100%) of the bleach intensity, the remaining 9% should correspond to the ring-opening products. This, however, is not guaranteed, considering that even if no ring-opening products were present in the product mix, the TRPES signal intensity at 9.1 eV (the first ionization band of furan) would decrease due to the presence of the 5-oxabicyclo[2.1.0]pent-2-ene molecule, which has the first ionization band (9.6 eV) shifted to higher ionization energies. Hence, the conclusion that the furan photoreaction leads to ca. 9% of the ring-opening photoproducts [9] may be not fully supported by the evidence.

### 3.3. NAMD Simulations of the $\pi \pi^{*}$ Relaxation

**Figure 6:**
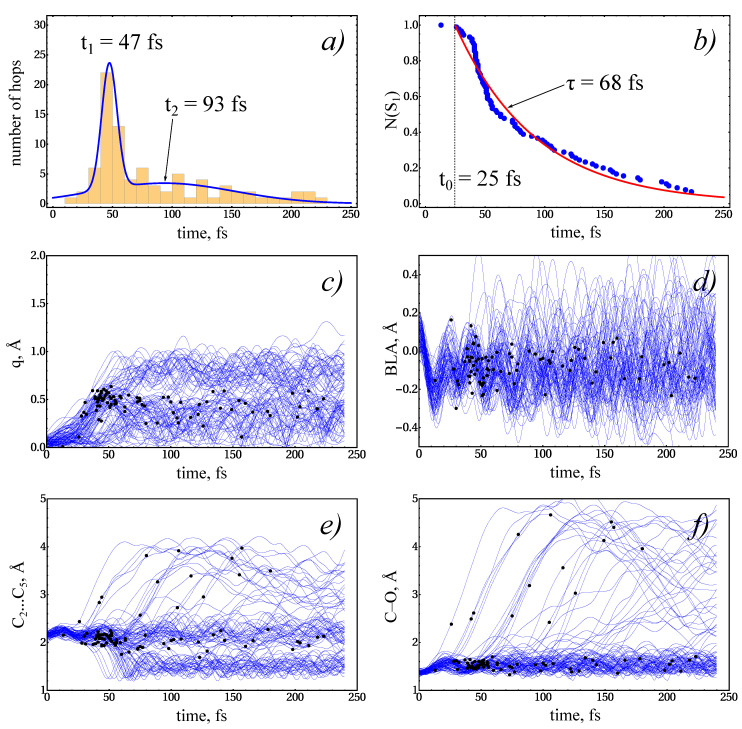
Simulations of the non-adiabatic dynamics of the $\pi \pi^{*}$ excited-state decay. (**a**) Distribution of the hop times. The blue line shows a bi-Gaussian fit of the distribution. (**b**) Excited-state population as a function of time. The red line shows a monoexponential fit with the latency $t_{0}$ and decay constant τ. (**c**) Plot of the Cremer–Pople puckering amplitude *q* for all the trajectories as a function of time. (**d**) Plot of the BLA distortion for all the trajectories as a function of time. (**e**) C${}_{2}$...C${}_{5}$ distance as a function of time. (**f**) The longest C–O distance as a function of time. In panels (**c**–**f**), the black dots show the surface hops.

The NAMD simulations of the non-adiabatic decay of the $\pi \pi^{*}$ excited state were performed using the DISH-XF/SSR-BH&HLYP/6-311G** method, as described in the Section 2.2. One hundred trajectories were started from the initial geometries and velocities obtained by sampling the Wigner function (T = 300 K) built at the S${}_{0}$ equilibrium geometry. The results of the NAMD simulations are summarized in Figure 6 and in Table 1.

From the previous inspection of the excited-state adiabatic PESs, the following geometric distortions can characterize the trajectories: The puckering distortion is needed to reach the ring-puckering CI. Here, we use the Cremer–Pople ring puckering coordinates [87] to describe this distortion. The puckering amplitude *q* at the S${}_{0}$ equilibrium geometry is zero, which characterizes a planar five-membered ring. At the ring-opening CI${}_{\mathrm{ropn}}$ geometry, the ring remains nearly planar, and q=0.007 Å. At the ring-puckering CI${}_{\mathrm{puck}}$, *q* becomes 0.412 Å, and at the equilibrium geometry of 5-oxabicyclo[2.1.0]pent-2-ene, it reaches a value of 0.795 Å. Hence, it may be expected that the trajectories propagating along the ring-puckering path will display large puckering amplitudes.

The formation of the C${}_{2}$–C${}_{5}$ bond in 5-oxabicyclo[2.1.0]pent-2-ene is monitored with the respective distances. In furan, the C${}_{2}$...C${}_{5}$ distance is 2.167 Å, and it shortens to 1.444 Å in the bicyclic molecule. The ring-opening is conveniently monitored by the length of the two C–O bonds of furan. At the equilibrium geometry, both C–O bonds have a distance of 1.347 Å, and at the CI${}_{\mathrm{ropn}}$ geometry, one of the distances stretches to 2.575 Å (see Figure 1). As the S${}_{2}$← S${}_{0}$ transition at the FC geometry results in weakening of the lateral C–C π-bonds and in strengthening of the C${}_{3}$–C${}_{4}$π-bond (see the respective Dyson orbitals in Figure 4), the $\pi \pi^{*}$ excitation is expected to result in a bond-length alternation (BLA) distortion, which is defined as the difference between the length of the formally single C${}_{3}$–C${}_{4}$ bond and the average length of the formally double C${}_{2}$=C${}_{3}$ and C${}_{4}$=C${}_{5}$ bonds of furan ring.

As seen in Figure 6, at the early time of t≲ 50 fs of the photoreaction, the majority of the trajectories undergo substantial ring puckering (see Figure 6c), and only a few (three) trajectories follow the ring-opening path, as shown in Figure 6f. The prevalence of the ring-puckering path was already anticipated when inspecting the adiabatic reaction paths in Section 3.1. At ca. 40–50 fs, a large fraction of the surface hops to the S${}_{0}$ state occur, as shown in Figure 6a, and nearly all of them at the ring-puckering CI. During this time period, the trajectories display pronounced synchronicity of the BLA displacement, especially in the first ca. 25 fs. The synchronous BLA displacement occurs predominantly at the almost planar ring geometry during the latency time seen in Figure 6b.

After ca. 20–25 fs, the initial vibrational excitation that was mostly localized in the normal modes describing the BLA distortion begins to proliferate to other vibrational modes, and particularly to the ring-puckering modes. When the ring-puckering amplitude *q* reaches the range of values around 0.5 Å, the large part of the trajectories hop into the S${}_{0}$ state (Figure 6c), where they split into two streams, one toward the bicyclic geometry and one back to the initial monocyclic furan geometry (Figure 6c,e). The trajectories that did not make it on the first approach to CI${}_{\mathrm{puck}}$ remain on the excited-state PES, where they roam in the search for a CI. Overall, 86% of the trajectories pass through the ring-puckering CI${}_{puck}$, and only 14% follow the path through CI${}_{ropn}$. The surface hops occur within a prolonged time interval and, at the end of the simulation window (240 fs), five out of one hundred trajectories still remain in the excited state.

The distribution of the hop times and the excited-state population (which is defined as a fraction of the total number of the trajectories residing in the excited state at a given instance of time) were fitted by a bi-Gaussian function $G\left(t\right)=c\;\frac{1}{\sigma_{1}\sqrt{2\pi }}\;e^{-{\left(t-\mu_{1}\right)}^{2}\;/\;2\sigma_{1}^{2}}+\left(1-c\right)\;\frac{1}{\sigma_{2}\sqrt{2\pi }}\;e^{-{\left(t-\mu_{2}\right)}^{2}\;/\;2\sigma_{2}^{2}}$ and a mono-exponential function $f\left(t\right)=e^{-\left(t-t_{0}\right)\;/\;\tau }$, respectively. The results of the fitting are reported in Table 1, where the margins of error were evaluated by bootstrapping [88] using 10${}^{4}$ random replicas of the initial datasets. The S${}_{2}$ lifetime (here, we label the $\pi \pi^{*}$ state as S${}_{2}$, although it becomes the lowest adiabatic excited state soon after the start of the photoreaction [4]) produced by the analysis of the DISH-XF/SSR-BH&HLYP/6-311G** trajectories is 93 ± 9 fs, which seems to be in agreement with the results of the previous NAMD simulations of furan (no precise decay parameters were reported) [4,9].

What seems to be at odds with the previous NAMD simulations is the distribution of the products. Although the current simulation time (240 fs) is too short to reach an equilibrium distribution, nevertheless, some conclusions may already be drawn from these results. At the end of the simulations, 43% of the trajectories fall back to the initial conformation (furan), 31% undergo electrocyclization to 5-oxabicyclo[2.1.0]pent-2-ene, and 26% undergo ring opening. (Note that only 14% of the trajectories pass through the ring-opening CI${}_{ropn}$, such that a large fraction of the ring openings occur on the S${}_{0}$ surface.) The NAMD simulations by Hua et al. [19] and Oesterling et al. [20], who used the CASSCF method, predicted that the majority of the trajectories undergo ring opening. The simulations by Fuji et al. [4] and Adachi et al. [9], who used the linear-response TDDFT methodology, predicted that only a tiny fraction (ca. 6%) of the trajectories undergo ring opening. Both sets of simulations seem to be flawed, although for somewhat different reasons. The CASSCF method lacks the dynamic electron correlation and, hence, it overestimates the vertical excitation energies (by a few eV) and underestimates the strength of the chemical bonds. Hence, the C–O bonds may appear too weak in the CASSCF description, and the fraction of the ring openings may be strongly overestimated. The TDDFT formalism suffers from two serious drawbacks: It cannot properly describe the topology of the conical intersections, especially with the S${}_{0}$ state [21,22,23], and it cannot describe bond dissociation in the ground electronic state [26,89]. Hence, predictions of TDDFT regarding the excited-state relaxation dynamics should not be trusted, and this method should not be used for the NAMD simulations. The SSR method used here can perfectly do both the proper description of the conical intersections [22,23,27] and the accurate description of the bond dissociation in the S${}_{0}$ state [24,26].

### 3.4. TRPE Spectra of Excited Furan

**Figure 7:**
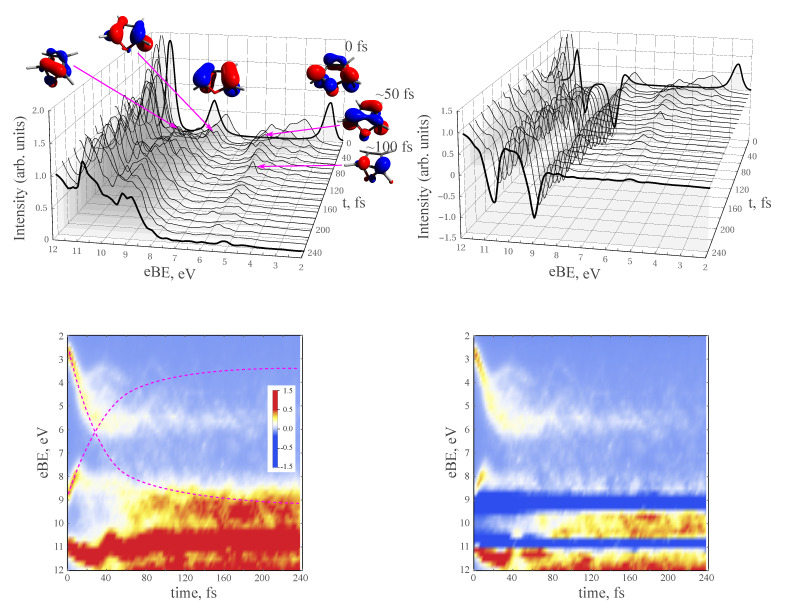
TRPE spectra of excited furan generated using Equation (7) with $\lambda_{\varepsilon }$ = 0.1 eV and $\lambda_{t}$ = 1.2 fs. Upper-left panel: The TRPE spectrum shown as a sequence of PE spectra at several time slices. The insets show the two lowest-energy Dyson orbitals of furan at the indicated instances of time. Upper-right panel: The difference TRPE spectrum obtained by subtracting the S${}_{0}$ furan PE spectrum from the TRPE spectrum in the upper-left panel. Lower panels: 2D representation of the TRPE spectra in the upper panels. The magenta dashed lines in the lower-left panel are guides for an eye indicating the directions of transformation of the two lowest Dyson orbitals.

Based on the results of the NAMD simulations and using Equation (7) with $\lambda_{\varepsilon }$ = 0.1 eV and $\lambda_{t}$ = 1.2 fs, the TRPE spectra of the excited furan were calculated. The spectra are displayed in Figure 7, where the full TRPE spectrum and the difference TRPE spectrum are shown in the left and the right panels, respectively. The latter spectrum was obtained by subtracting the S${}_{0}$ PE spectrum of furan from the full TRPE spectrum. The lower panels in Figure 7 show the 2D projections of the TRPE spectra in the upper panels.

At the start of the photoreaction, the TRPES is identical to the static PE spectrum of furan in the $\pi \pi^{*}$ excited state; see Figure 4. Following the initial geometric transformation of the excited furan, the peaks occurring at 2.7 eV ($3b_{1}$) and 8.7 eV ($1a_{2}$) undergo a rapid relaxation in converging directions; see also the dashed magenta lines in the lower-left panel of Figure 7. The transformation of the corresponding Dyson orbitals is displayed in the insets in the upper-left panel of Figure 7. This transformation is consistent with the transformation of Dyson’s orbitals along the adiabatic MEPs shown in Figure 2, which is typical for symmetry-forbidden reactions. In particular, the $1a_{2}$-symmetric (8.7 eV at *t* = 0 fs) Dyson orbital shifts to lower eBE and loses intensity as it becomes depopulated during the non-adiabatic relaxation in the S${}_{0}$ state. The $3b_{1}$-symmetric Dyson orbital shifts to higher eBE as a consequence of moving towards the puckering CI, and gains intensity as the respective natural orbital becomes populated with two electrons.

In Figure 7, the orbitals typical for the ring closure to 5-oxabicyclo[2.1.0]pent-2-ene are displayed, as this reaction proceeds through the dominant ring-puckering channel. The other dominant reaction, the return to the original furan conformation, proceeds through both ring-puckering (major) and ring-opening (minor) channels. However, the orbital transformation during the back reaction is trivial; the $3b_{1}$-orbital becomes depopulated, and the $1a_{2}$ orbital becomes doubly populated. The initial orbital relaxation appears to be identical for both the forward and the backward reactions. The ring-opening is a minor channel (only 14% of the trajectories pass through CI${}_{ropn}$; see above), and exclusion of the trajectories passing through the ring-opening CI does not result in discernible changes of the TRPE spectrum.

The difference TRPE spectrum reproduces the main features of the experimentally measured TRPES from [9]. The deep trenches at ca. 9 and 11 eV correspond to the bleach of the ground-state PES, and the intensity of these bands gradually recovers during the photoreaction. There is also a positive buildup of the intensity between the two troughs, which is associated with the formation of the vibrationally hot products of the photoreaction.

**Figure 8:**
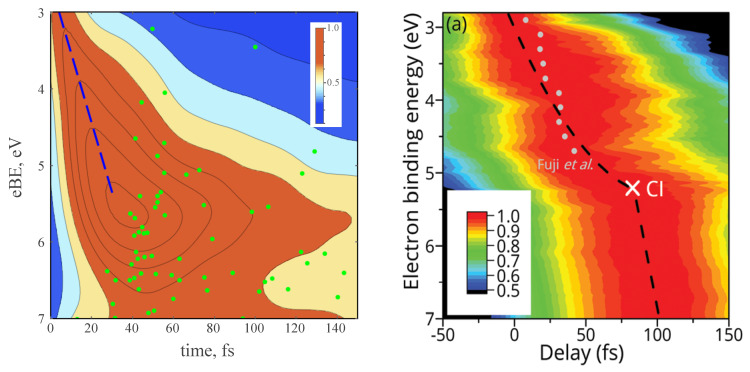
Low-energy part of the TRPE spectrum of excited furan. Left panel: The TRPE spectrum obtained with Equation (7) with $\lambda_{\varepsilon }$ and $\lambda_{t}$ adopted from [9] ($\lambda_{\varepsilon }$ = 0.5 eV and $\lambda_{t}$ = 10 fs). The green dots in the left panel show the surface hops to the S${}_{0}$ state. The blue dashed line in the left panel shows the intensity shift at the early time of the photoreaction. The rate of the shift obtained here is 0.091 eV/fs, which can be compared with 0.094 eV/fs obtained in [5]. The right panel shows the TRPE spectrum obtained by Adachi et al. and reproduced from [9] with permission (copyright of the Royal Chemical Society, 2018). See Figure 3a of [9] for more details. The superscript (a) in the right panel originates from Figure 3a of [9].

As regards the low eBE peaks, their intensity is difficult to compare with the TRPE spectrum reported by Adachi et al. [9] for at least two reasons: (i) The theoretical intensity is approximated by the norm of the respective Dyson orbitals, and (ii) the experimental intensity in [9] was recalibrated to make certain features more pronounced; see Figure 3a of [9] and the accompanying text. Nevertheless, a comparison with the TRPES reported in Figure 3a of [9] was attempted by redrawing the low-energy part of the theoretical spectrum with the broadening parameters adopted by Adachi et al. [9], i.e., $\lambda_{\varepsilon }$ = 0.5 eV and $\lambda_{t}$ = 10 fs. The resulting theoretical spectrum is shown in the left panel of Figure 8, where it is compared with the experimental TRPES in Figure 3a of [9].

Although there are certain visual differences between the theoretical and experimental TRPE spectra, there is a vague agreement between the main features. At the start of the photoreaction, there is a rapid intensity shift, which is shown with the dashed blue line in the left panel of Figure 8. The theoretically estimated rate of the shift is 0.091 eV/fs, which is comparable with the experimentally measured rate of 0.094 eV/fs [5]. However, the theoretical spectrum does not seem to produce a kink on the intensity line; see the dashed black line in the right panel of Figure 8. Adachi et al. argued that the kink is a signature of the passage through a conical intersection. The kink was assigned [9] to an abrupt change in the potential energy slope as the molecule passed through the conical intersection.

**Figure 9:**
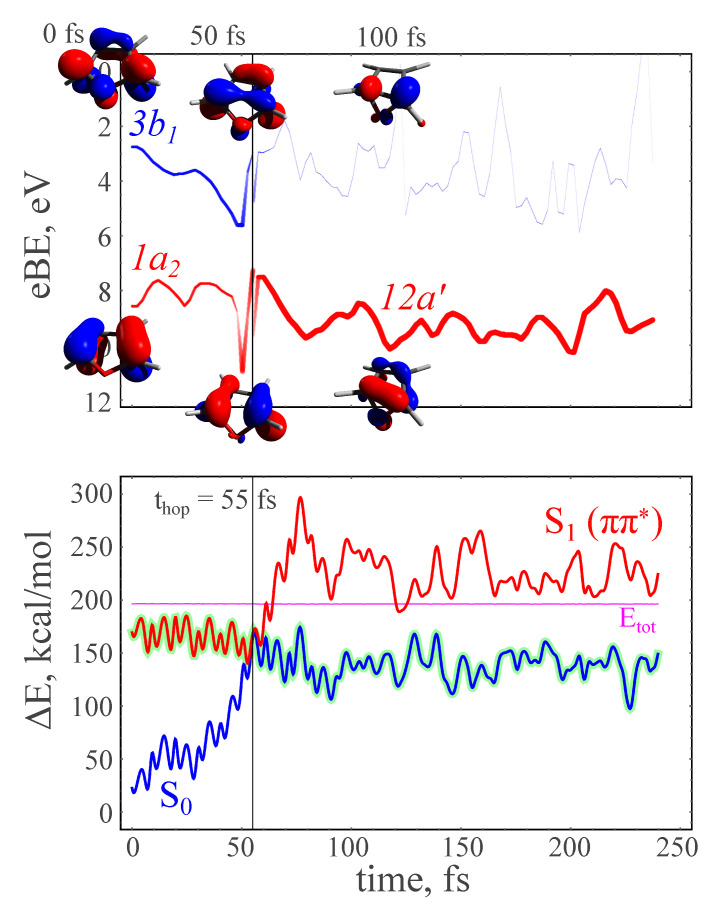
Exemplary trajectory (ring closure to 5-oxabicyclo[2.1.0]pent-2-ene) that illustrates time variation of the energies of the two lowest Dyson orbitals (upper panel) and the total energies (with respect to the S${}_{0}$ equilibrium geometry) of the ground and excited states (lower panel). The thickness and the opacity of the lines in the upper panel display the norms (probability of ionization) of the respective Dyson orbitals. The vertical line indicates the time of the surface hop to the S${}_{0}$ state (55 fs). The horizontal line in the lower panel shows conservation of the total energy, i.e., the electronic + the nuclear kinetic energy. The running PES is highlighted.

However, the plausibility of this argument seems to be questionable. Indeed, as seen in the left panel of Figure 8, passage through conical intersections occurs within a broad range of energies and times; see the green dots in the theoretical spectrum. This alone would be sufficient to smear out any sharp features resulting from the individual surface hopping events. Besides that, as shown in Figure 9, the potential energy of the ground and excited states does not indicate any abrupt change in the slope in the vicinity of the conical intersection. Figure 9 displays the potential energies and the ionization potentials (Dyson’s orbital energies) along an individual NAMD trajectory, which leads to the ring closure to 5-oxabicyclo[2.1.0]pent-2-ene. The main features of other trajectories are similar to those of the one shown in Figure 9. It is noteworthy that the potential energy has a pronounced oscillatory component as a result of the vibrational motion of the atoms during the dynamics. The oscillations are less pronounced in the ionization potentials shown in the upper panel of Figure 9. However, the transition through the conical intersection does not result in a change in the slope of the ionization potential that would manifest itself as a kink in the TRPE spectrum. Hence, we have to conclude that, most likely, the kink in the observed TRPE spectrum of excited furan results from factors different from the passage through conical intersections.

In the molecular valence region, the energies and the norms of Dyson’s orbitals depend strongly on the molecular geometry. Hence, the TRPE spectrum in the valence region depends not only on the occupations of the ground and excited states, but also on the geometric evolution of the molecule during the dynamics [34,35]. Although such a spectrum contains a wealth of information about the reaction dynamics, its interpretation is not at all straightforward, and may require an input from other experimental techniques and theoretical calculations. However, the core electronic levels, e.g., the K-shell electrons, do not directly partake in the orbital transformations during the dynamics; however, they are quite sensitive to the charge state and the chemical environment of the element [90]. As passage through conical intersection often results in a noticeable change in the density of the valence electrons, which is manifested, e.g., in the atomic electron density fluctuations, monitoring the core levels may provide more reliable signatures of the passage.

**Figure 10:**
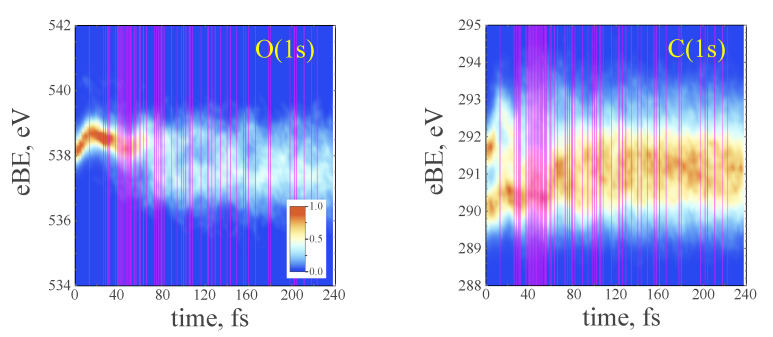
Simulated TRPE spectra of furan in the energy region of the 1s-electrons of oxygen (**left panel**) and the 1s-electrons of carbon atoms (**right panel**). The vertical magenta lines show the instances of passage through conical intersections.

**Figure 11:**
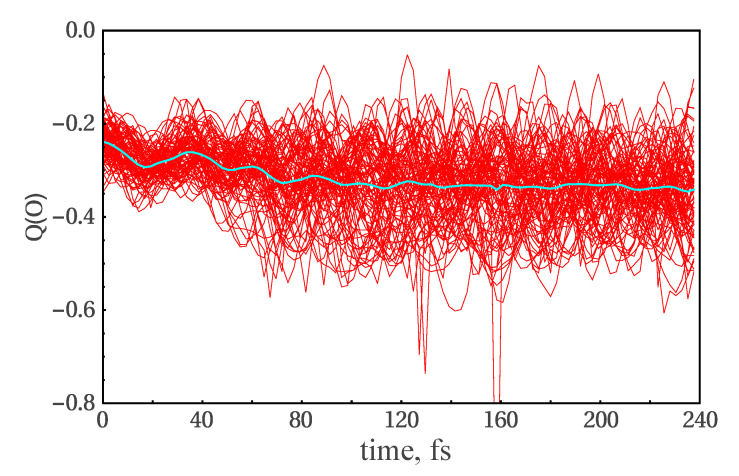
Mulliken charge on the O atom along the trajectories; the blue line shows an average over the trajectories.

Indeed, as shown in Figure 10 (in Figure 10, the positions of the O(1s) and C(1s) peaks at t=0 fs agree with the ESCA spectra [91,92]; In the ESCA spectra [91,92], the C(1s) peaks occur at 290.3 ± 0.1 eV and 291.5 ± 0.1 eV, and the O(1s) peak occurs at 539.4 ± 0.1 eV; a slight shift, ca. 1.2 eV, of the calculated O(1s) peak to lower binding energies can be explained by the absence of the relativistic corrections in the present calculations), the K-shell of oxygen is quite sensitive to the surface hop events. At the start of the trajectories, the PE signal of the 1s-electrons of oxygen displays high intensity, with the energy shifting towards higher eBE. The energy shift is likely caused by the variation of the local charge density on the O atom during the dynamics. Initially, as furan slides down the excited-state PES towards CI${}_{\mathrm{puck}}$ (the dominant reaction channel), the absolute value of the Mulliken charge on the O atom increases coherently along the trajectories; see Figure 11. After the first ca. 40 fs, distribution of the atomic charges becomes broader. This is caused by the vibrational motion becoming more vigorous as the nuclear kinetic energy increases; see, e.g., the difference between the running surface potential energy and the total energy $E_{tot}$ in Figure 9. Furthermore, when the S${}_{1}$→ S${}_{0}$ surface hops occur, the trajectories take different paths, as the molecule is in a vibrationally hot state, and the atomic charges begin to vary randomly and are spread across a broad range of values; see Figure 11. Hence, the positions of the oxygen K-shell energy levels disperse across a range of values that is ca. 4 eV wide, and the PE signal loses intensity; see Figure 10.

The time profile of the PE intensity of the oxygen K-shell nicely correlates with passage through conical intersections. Interestingly, the K-shell energy levels of carbon atoms do not display a clear correlation with the surface hop events. Although for the individual carbon atoms, the same trends as for the oxygen may hold, superposition of the signals of the individual atoms results in a broad band of approximately constant intensity. It is therefore not obvious whether monitoring the PE intensity at the C K-shell binding energies can provide information on the photoreaction dynamics.

The parameters of the photoreaction can be obtained from the time profiles of the PE signals at the binding energies corresponding to the first ionization band of the S${}_{0}$ state of furan (the main product) and the K-shell of oxygen. Indeed, the recovery of the ground-state signal should show sufficiently good correlation with the time spent by the molecule in the excited state. This can be seen, e.g., in Figure 9, where the ionization potential of furan settles in the energy range (∼8–9 eV) typical for the S${}_{0}$ state soon after passing through the conical intersection. Therefore, fitting the PE intensity at these binding energies may provide a good estimate of the excited-state lifetime, i.e., the population dynamics.

**Figure 12:**
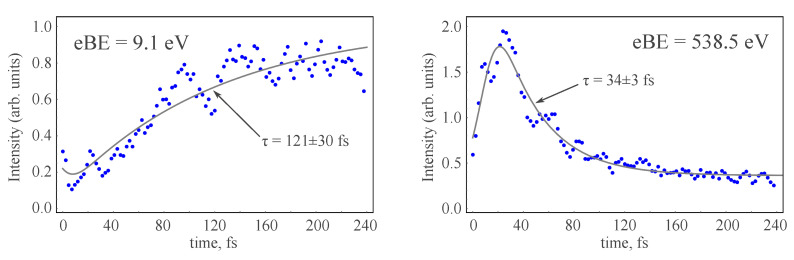
Time profile of TRPES intensity at eBE = 9.1 eV (the first ionization maximum in S${}_{0}$ state) and 538.5 eV (the K-shell of O). Solid lines are fitted using the exGaussian function. The fitted exponential decay constants τ are also shown. The other parameters of the exG function (Equation (8)) are: (**left panel**) *A* = −1.067 ± 0.224, *B* = 1.015 ± 0.088, σ = 15 ± 18 fs, μ = −17 ± 24 fs, $R^{2}$ = 0.982; (**right panel**) *A* = 2.363 ± 0.128, *B* = 0.363 ± 0.018, σ = 10 ± 1 fs, μ = 8 ± 1 fs, $R^{2}$ = 0.985.

Figure 12 shows the results of fitting the PE intensity at eBE = 9.1 eV and 538.5 eV using an exGaussian function, i.e., the convolution of a Gaussian and an exponential function:

(8) $$\begin{array}{cc} \; & exG(x;A,B,\tau ,\sigma ,\mu )\\ \; & =A\;\frac{1}{\sigma \;\sqrt{2\pi }}\;\int_{0}^{\infty }\;e^{-y\;/\;\tau }\;e^{-{\left(x-y-\mu \right)}^{2}\;/\;2\sigma^{2}}dy+B\\ \; & =\frac{A}{2}\;e^{-1\;/\;\tau (x-\mu -\sigma^{2}\;/\;2\tau )}\;\left(1+Erf\left[x-\mu -\sigma^{2}\;/\;2\tau \;/\;\sigma \sqrt{2}\right]\right)+B\;, \end{array}$$

where *A* and *B* are the amplitude and the background constant, τ is the exponential decay parameter, and σ and μ are the variance and the offset of the Gaussian distribution function. The exG function is often used when fitting the experimental spectra, as it helps to incorporate the finite instrument response time [7,8,9].

The fitted decay constants are in reasonable agreement with the population dynamics (the signal at eBE = 9.1 eV) and with the time of surface hops (eBE = 538.5 eV). Thus, $\tau_{9.1}=121\pm 30$ fs from the simulated TRPE spectrum falls within the error bars of the excited-state population decay constant $\langle \tau_{S_{2}}\rangle =93\pm 9$ fs. This value is also in reasonable agreement with the ground-state recovery constant of ca. 110 fs obtained by Adachi et al. [9]. The first maximum in the distribution of the surface hops in Figure 6a occurs at 47 ± 2 fs (see Table 1), which is close to the $\tau_{538.5}=34\pm 3$ fs obtained from the TRPE spectrum in Figure 10. It can therefore be suggested that TRPE spectroscopy be used in combination with the time-resolved XPS (trXPS) method [12] to refine the description of the excited-state dynamics.

## 4. Conclusions

In this work, we investigated the dynamics of furan excited in the $\pi \pi^{*}$ state (S${}_{2}$ at the FC geometry) using non-adiabatic molecular dynamics simulations and analysis of the adiabatic potential energy surfaces and the theoretically estimated TRPE spectra. According to the DISH-XF/SSR-BH&HLYP/6-311G** NAMD simulations, the relaxation of the excited state of furan occurs predominantly through the ring-puckering channel; ca. 86% of all NAMD trajectories passed through the ring-puckering conical intersection. However, the final distribution of the reaction products is determined by the events occurring in the S${}_{0}$ state after relaxation from the excited state. Thus, after 240 fs (the duration of the NAMD simulations in this work), 43% of the trajectories fell back to the initial conformation (furan), 31% underwent electrocyclization to 5-oxabicyclo[2.1.0]pent-2-ene, and 26% ended up in various open-ring conformations. The determination of the exact distribution of possible reaction products requires much longer simulations, probably on the order of the gas-phase collision rate, which is definitely beyond the scope of the present work.

The characteristics of the photodynamics obtained in this work seem to be in reasonable agreement with the experimental observations. Thus, Adachi et al. [9] carried out gas-phase TRPES measurements (the probe photon energy 14 eV) and determined that the photoreaction proceeds predominantly through the ring-puckering channel. The ground-state recovery constant obtained from the experimental TRPE spectra was ca. 110 fs (no error bars were reported in [9]), which agrees reasonably with the theoretical excited-state lifetime of 93 ± 9 fs and with the S${}_{0}$ recovery constant of 121 ± 30 fs obtained from the theoretical TRPE spectra. The early dynamics of the TRPES signal estimated in this work agree with the experimental measurements, such as those reported by Fuji et al. [4] and Spesyvtsev et al. [5]. For example, the rate of change of the PE intensity estimated from the theoretical TRPE spectra is 0.091 eV/fs and the experimentally measured rate is 0.094 eV/fs [5]. Consequently, it can be assumed that the present theoretical simulations reproduce the dynamics of furan excited in the $\pi \pi^{*}$ state sufficiently well.

The main difference between the present theoretical simulations and the experimental observations reported by Adachi et al. [9] is that no discernible kink in the PE intensity was observed in the theoretical TRPE spectra; see Figure 8. Adachi et al. [9] observed a kink in the rate of change of the PE intensity (see Figure 8), which they associated with passage through conical intersections. However, the theoretical simulations show that the non-adiabatic population transfer through conical intersections occurs within a sufficiently broad range of electron binding energies and propagation times. Hence, any sharp features that may result from an individual event should be smeared out and masked in an ensemble of a large number of trajectories. It is therefore plausible to assume that the transitions through conical intersections should have different manifestations in the measured TRPE spectra from those of kinks of the PE intensity.

The PE signal intensity of the valence electrons experiences a strong influence from the geometric changes that the molecule undergoes during the dynamics [34,35]. Although these variations carry a wealth of information about the photoreaction, their analysis requires a substantial input from theory, and the features observed in the valence TRPE spectrum may not be directly related with the excited-state population dynamics, e.g., the excited-state lifetime or the time of passage through conical intersections. Regarding the photoreaction mechanism, the main conclusions that can be drawn from the present study are:

- The time of transition through conical intersections is best represented by the time profile of the PE (or the photo absorption) intensity of the deep core electrons, e.g., the K-shell electrons. The latter can be obtained by the trXPS [12] or the trNEXAFS [14,15] techniques.
- The excited-state lifetime can be estimated from the dynamics of the recovery of the ground-state PE intensity.

Regarding the former statement, the core electrons do not partake in the chemical transformations; however, their energy levels are sensitive to the chemical environment of the element. In particular, the electrostatic fields resulting, e.g., from the valence electron density on the individual atoms in the molecule shift the K-shell energy levels of light elements [90]. As the valence electron density experiences a rapid change during the passage through conical intersections, the K-shell levels shift, and this leads to a decay of the PE (or the photo absorption) intensity at the given electron binding energy. For example, monitoring the time profile of the PE intensity at the eBE = 538.5 eV (K-shell of oxygen) in the theoretical TRPE spectrum reveals that its decay constant (34 ± 3 fs) agrees sufficiently well with the time of the most intense transitions through conical intersections (47 ± 2 fs). Initially, the PE intensity of the K-shell of O is high and follows variations of the atomic electron density, which undergoes a coherent displacement towards greater magnitudes during the early dynamics. As the first transitions to the S${}_{0}$ state occur, the variations of the atomic electron density become less coherent, and this leads, i.a., to the decay of the K-shell PE intensity at the given eBE; see Figure 10, Figure 11 and Figure 12.

The recovery of the ground-state PE intensity in the valence region is another characteristic, the time profile of which can provide information on the excited-state lifetime. Although, after the transition to the ground state, the molecule is formed in a vibrationally hot state, its electronic characteristics, e.g., the first ionization potential, are sufficiently rapidly restored to the range of values typical for the ground state. Thus, as shown in Figure 9 for an individual trajectory, the energy and intensity of the first ionization band reach the range of values expected for the S${}_{0}$ state (i.e., around 9.1 eV) within 10 fs after the passage through the ring-puckering CI. This behavior is also typical for other trajectories. At the same time, the energy of the outermost Dyson orbital of the excited furan shifts very rapidly during the dynamics preceding the non-adiabatic transition, and the decay of the PE intensity in this region of energies does not seem to carry information on the time of passage through conical intersections; see Figure 9. Hence, the ground-state recovery seems to be a sufficiently reliable indicator of the excited-state lifetime. The decay constant of the recovery of the ground-state PE intensity from the theoretical spectrum is 121 ± 30 fs, which is in agreement with the theoretical excited-state lifetime of 93 ± 9 fs, as well as with the experimental PE intensity recovery time constant of ca. 110 fs [9].

The results of the present work supplement the previously obtained evidence on the significance of the structural (geometric) evolution for the time decay of the outermost peaks in the TRPE spectra of excited molecules [34,35]. The characteristics of the TRPE (and/or trXPS and trNEXAFS) spectra suggested here can reveal information on the time of occurrence of the important events during the excited-state dynamics, such as the transfer through conical intersections and the excited-state lifetime, thus complementing the range of conceptual tools used for the interpretation of the TRPES measurements.

## Figures and Tables

**Table 1:** Parameters of the bi-Gaussian fit of the hop times and the mono-exponential fit of the excited-state populations.

hop times ^1^	*c*	$\mu_{1}$, fs	$\sigma_{1}$, fs	$\mu_{2}$, fs	$\sigma_{2}$, fs
	0.40 ± 0.08 ^2^	47 ± 2	6 ± 2	93 ± 18	59 ± 11
S${}_{2}$ decay ^3^	τ, fs	$t_{0}$, fs	$\langle \tau_{S_{2}}\rangle$, fs		
	68 ± 10	25 ± 3	93 ± 9		

^1^ a bi-Gaussian function $G\left(t\right)=c\;\frac{1}{\sigma_{1}\sqrt{2\pi }}\;e^{-{\left(t-\mu_{1}\right)}^{2}\;/\;2\sigma_{1}^{2}}+\left(1-c\right)\;\frac{1}{\sigma_{2}\sqrt{2\pi }}\;e^{-{\left(t-\mu_{2}\right)}^{2}\;/\;2\sigma_{2}^{2}}$ is used for fitting of the hop times; ^2^ margin of error determined by bootstrapping [88] with $10^{4}$ replicas; ^3^ a mono-exponential function $f\left(t\right)=e^{-\left(t-t_{0}\right)\;/\;\tau }$ is used for fitting of the excited-state populations.

## Data Availability

Not applicable.
